# Metal-Organic Frameworks-Based Sensors for the Detection of Toxins in Food: A Critical Mini-Review on the Applications and Mechanisms

**DOI:** 10.3389/fbioe.2022.906374

**Published:** 2022-05-26

**Authors:** Xiaoxu Xuan, Mengjie Wang, Sivakumar Manickam, Grzegorz Boczkaj, Joon Yong Yoon, Xun Sun

**Affiliations:** ^1^ Key Laboratory of High Efficiency and Clean Mechanical Manufacture, Ministry of Education, School of Mechanical Engineering, Shandong University, Jinan, China; ^2^ National Demonstration Center for Experimental Mechanical Engineering Education, Shandong University, Jinan, China; ^3^ Petroleum and Chemical Engineering, Faculty of Engineering, Universiti Teknologi Brunei, Bandar Seri Begawan, Brunei Darussalam; ^4^ Department of Sanitary Engineering, Faculty of Civil and Environmental Engineering, Gdańsk University of Technology, Gdańsk, Poland; ^5^ Advanced Materials Center, Gdansk University of Technology, Gdansk, Poland; ^6^ Department of Mechanical Engineering, BK21 FOUR ERICA-ACE Center, Hanyang University, Ansan, South Korea; ^7^ Department of Mechanical Engineering, The University of Hong Kong, Hong Kong, Hong Kong SAR, China

**Keywords:** metal–organic framework, food safety, sensors, toxin, synthesis

## Abstract

Using scientific technologies to detect toxins in food is significant to prevent food safety problems and protect people’s health. Recently, the rise of sensors has made rapid, efficient, and safe detection of food toxins possible. One of the key factors impacting the sensor’s performance is the nanomaterials employed. Metal-organic frameworks (MOFs), with high specific surface area, tunable composition, porous structure, and flexible properties, have aroused the interest of researchers. The applications of MOFs in detecting food toxins have seen remarkable success in the past few years. In this critical mini-review, the impact of various synthesis methods on MOFs’ properties is first presented. Then, the applications and mechanisms of MOFs-based sensors in detecting various toxins are summarized and analyzed. Finally, future perspectives, potential opportunities, and challenges in this field are discussed.

## Introduction

Nowadays, food safety has become a worldwide public concern as more and more people have suffered from foodborne illnesses in recent years (Wang et al., 2019; Tao et al., 2020). There have been numerous reported food poisoning incidents, leading to serious consequences (Xu et al., 2021; Tao et al., 2022). For example, in 2020, the government of the United States reported the detection of perfluoroalkyl sulfonate (PFAS) in the fast food packages. The intake of PFAS leads to liver damage, reduced fertility and even cancer. Another food poisoning incident occurred recently in Japan; in this case, more than 3500 teachers and students were present with diarrhoea caused by food poisoning. These outbreaks have put people’s lives at risk and caused a huge impact on local economies, thereby attracting great attention worldwide.

Based on the sources of the toxins, they can be roughly divided into the following catagories: i) veterinary drugs used to prevent bacterial infections and treat animal diseases, ii) pesticide utilized to increase agricultural production, iii) pathogens and mycotoxins due to improper packaging of food, spoilage and deterioration, and iv) heavy metals, illegal additives, and other contaminants related to illegal processing of food. These toxins could accumulate in food products and lead to health-related problems when consumed by the human beings (Cheng et al., 2021).

In order to realize the rapid detection of toxins and ensure food safety, various toxins detection techniques have recently been developed, including novel sensing methods such as luminescent, electrochemical, colorimetric, and surface-enhanced Raman scattering (SERS) methods. Different categories of sensors have been invented, and the key factors determining the selectivity, sensitivity, stability, and cost are the design and selection of sensing materials employed in the sensors (Fang et al., 2018).

Metal-organic frameworks (MOFs) are porous hybrid nanomaterials fabricated through the linkage of metal ions/clusters centers and organic ligands (Dutta et al., 2020; Liu et al., 2020; Dutta et al., 2021; Xuan et al., 2022). MOFs hold great practical potential for toxins detection in food (Pinar Gumus and Soylak, 2021), where the characteristics of porosities, tunable compositions, and diverse structures and functions are vital. Up to now, a large number of MOFs have been designed and synthesized for the detection of toxins in food (Hitabatuma et al., 2022). However, the successful fabrication of MOFs with good sensitivity, selectivity, and detection limit remains a great challenge (Wang et al., 2020; Li et al., 2022), as the mechanisms of commonly used synthesis methods are not clear.

This review summarises the controlled synthesis and the properties of MOFs beneficial to rapidly detecting toxins in food and the effective methods to ensure food safety monitoring. Then, the applications and mechanisms of MOFs-based sensors in detecting various toxins are analyzed. The future perspectives, potential opportunities, and challenges are also discussed. The present review will shed light on this research field and inspire readers to conduct intensive studies.

## Controlled Synthesis of MOFs for Food Safety Sensors

**FIGURE 1:**
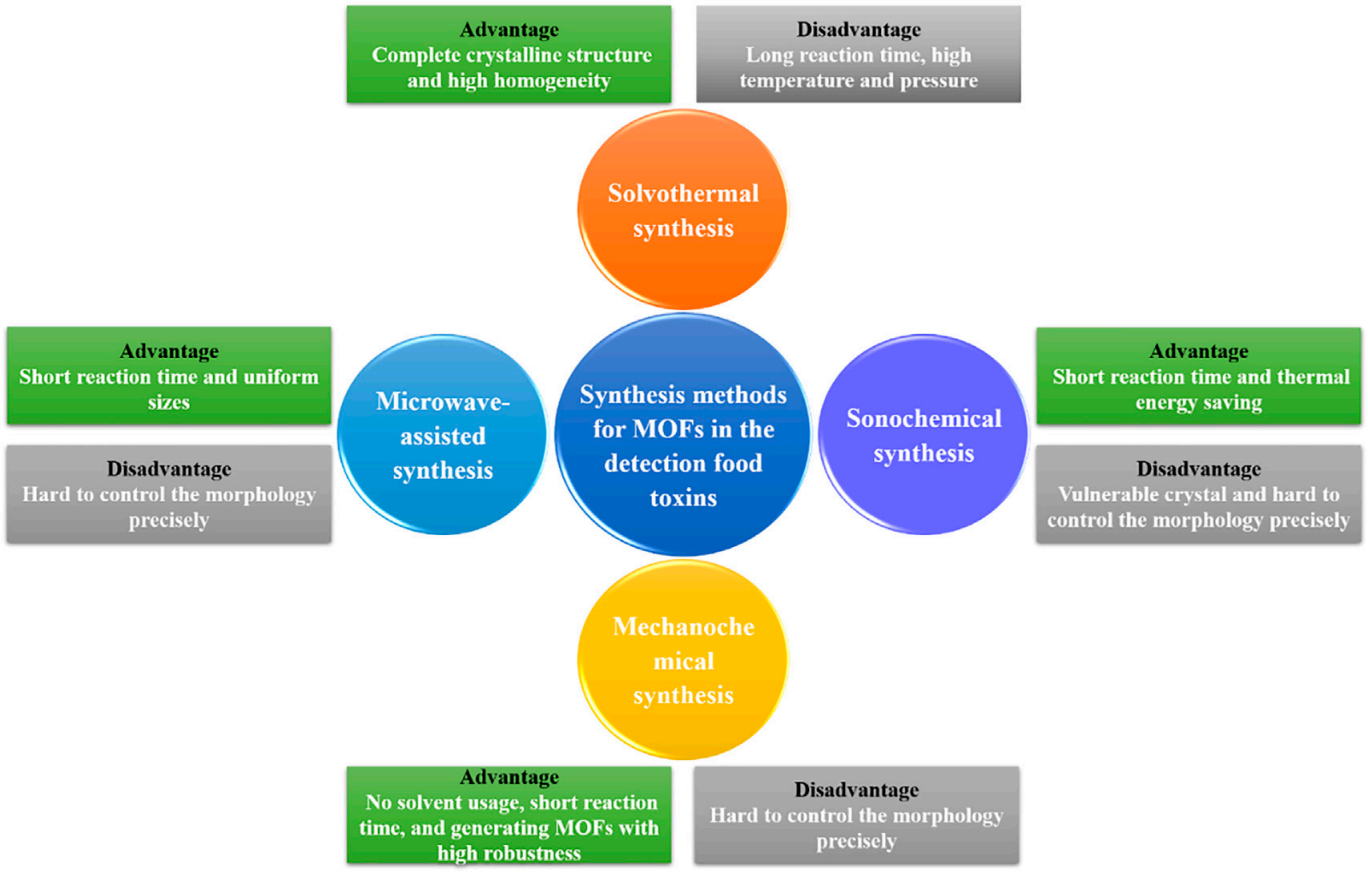
Commonly used MOFs synthesis methods (solvothermal, microwave-assisted, sonochemical, and mechanochemical) and their advantages and disadvantages.

Since the MOFs synthesis methods employed are the key factors dominating the effectiveness in toxins detection, it is important to have an in-depth understanding of the mechanisms underlying the synthesis methods. In a typical MOF fabrication process, the metal ions (copper, zinc, magnesium, zirconium, cobalt, etc.) are bridged with various organic ligands (carboxylates, tetrazolates, sulfonates, and cadmium) to form crystalline structures with linear, square planar, cubic, pyramidal, trigonal bi-pyramidal, tetrahedral, and octahedral shapes. The varieties of the metal centers, organic linkers, and solvents can lead to different topologies and food toxins detection properties of MOFs. Solvothermal, microwave-assisted synthesis, sonochemistry, and mechanochemistry are commonly used methods rising in recent years (Figure 1).

The solvothermal method is the most used among the above methods. Solvothermal reactions are generally carried out in autoclaves with excellent airtightness under high temperatures to achieve the rapid synthesis of MOFs. These reaction conditions are beneficial to the growth of MOFs crystals with unique morphologies that conventional methods cannot achieve. The most widely employed solvent is dimethylformamide (DMF). DMF can easily decompose into basic dimethylamine when heated, deprotonating the ligands and facilitating the combination of ligands and metal ions to form secondary building units (SBUs) of MOFs. Numerous researchers utilise DMF to ensure the rapid synthesis of MOFs. For example, (Yao et al., 2021) obtained Eu/Zr-MOFs nanomaterial to detect acrylamide by the solvothermal method using DMF solvent. The process was conducted in a stainless-steel high-pressure reactor with polytetrafluoroethylene lining and heated continuously at 120°C for 12 h. (Yang et al., 2021) reported the successful fabrication of NH_2_-MIL-125(Ti)-TiO_2_ composite for enhanced detection of oxytetracycline (OTC), which was achieved by using tetrabutyl titanate (TBT) and 2-aminoterephthalic acid (H_2_BDC-NH_2_) as the reagents and DMF and methanol as the solvents. The reaction was carried out in a Teflon-lined autoclave at 150°C for 20 h.

Since solvothermal reactions with conventional heating generally require a long time, alternative techniques such as microwave-assisted and sonochemical methods with shorter reaction times have emerged recently (Khan and Jhung, 2015). In microwave-assisted synthesis, solvent molecules with high dipole moments interact with electromagnetic radiation in dipole rotation and ionic conduction. The energy is then transferred from microwaves to solvent molecules to assist in crossing the activation energy of the reaction in the solvent, thereby largely shortening the reaction time. In the research by (Vakili et al., 2018), microwave-assisted synthesis of Zr-MOFs only required a reaction time of 2–2.5 h, much lower than conventional solvothermal synthesis (normally 24 h). Moreover, the homogeneous effects of microwaves can create a uniform seeding condition (Vinu et al., 2017), and by adjusting the microwave energy intensity, the nucleation and crystal growth rate can be easily controlled, enabling the size-controlled synthesis of MOFs. (Liu et al., 2017) reported the successful microwave synthesis of micron and nanometer-sized γ-cyclodextrin MOFs (γ-CD-MOFs) for the first time. The BET surface area of the as-synthesized γ-CD-MOFs (40–500 μm) reached 1,002 m^2^ g^−1^. The morphology of MOFs can also be tuned. Using microwave-assisted coordination modulation, (Sakata et al., 2012) altered the morphology of Zn_2_(ndc)_2_(dabco) [ndc: 1,4-naphthalenedicarboxylate; dabco: 1,4-diazabicyclo (Tao et al., 2020Tao et al., 2020Tao et al., 2020) octane] from large micron-sized cubic crystals to nano-sized rods by changing the quantity of modulator added to the reaction mixture.

In the sonochemical synthesis, the sonochemical effect resulting from bubble collapse under ultrasonic wave irradiation (Suslick et al., 1986; Xu et al., 2013; Sun et al., 2021a) can significantly enhance the solvothermal reaction rate. Its main advantage is that it can concentrate the diffuse energy of sound into a set of “super conditions” (∼5,000 K and ∼1,000 bar) to produce strengthened nanomaterials from precursors dissolved in solution (Suslick et al., 1999; Sun et al., 2022). Therefore, the sonochemical method reduces reaction time and energy consumption compared with microwaves (Sun et al., 2021b). In addition, the powerful shock waves and microjets generated during the reaction can effectively mix the solvents. In 2008, (Qiu et al., 2008) first reported utilizing ultrasound to synthesize fluorescent microporous MOFs. Since then, many research works on applying the sonochemical method in MOFs fabrication have been conducted. (Haque et al., 2010) performed a kinetic study to determine the reaction rates of conventional, microwave-assisted, and sonochemical methods. The average reaction time for the above methods to obtain the MIL-53(Fe) product is 1.5–3 days at 70–80°C, 1.5–2.5 h at 60–70°C, and 0.5–1 h at 50–70°C, respectively. Similar to microwaves, the sonochemical synthesis can precisely control the MOFs’ size. (Wiwasuku et al., 2020) used the sonochemical route to fabricate a microscale Zn(II)-MOF with dual Lewis basic sites for fluorescent turn-on detection of Al^3+^ and methanol. The employment of sonochemical strategy greatly reduced the nanoparticles sizes and easily enabled size-control synthesis of MOFs.

Unlike the methods mentioned above, mechanochemical synthesis does not require any solvent. In 2006, (Pichon et al., 2006) proposed the first solvent-free synthesis of a microporous MOF [Cu(INA)_2_] (INA = isonicotinic acid). The Cu(INA)_2_ exhibited robust 3-dimensional connectivity and was obtained quantitatively by grinding together copper acetate and isonicotinic acid for 10 min without any heating. The reaction time in mechanochemical synthesis is much shorter than other methods. (Liu et al., 2022) synthesized an X@HKUST-1(X = imidazole, 1,2,4-1H-triazole, and tetrazole) through a rapid mechanochemical synthesis in only 5 min at room temperature. The X@HKUST-1 showed a similar structure with the solvothermal method synthesized HKUST-1. Due to ease of use, low energy consumption, large production capacity, mechanochemical synthesis has great potential in large-scale productions. (Gao et al., 2021), reported a mechanochemical method for the rapid and scalable production of two pillared-layer MOFs, namely [Zn_2_(atz)_2_(ipa)] (Zn-atz-ipa) and [Zn_2_(datz)_2_(ipa)] (Zn-datz-ipa). This mechanochemical method could be scaled up to 100 mmol magnitudes with single-pass production up to 20 g.

Although all the synthesis methods presented above have been well demonstrated to be effective in fabricating MOFs with needed structures, it is important to choose an appropriate strategy and verify its effectiveness in practical applications. In addition, to meet the need to detect food toxins, combining two or three methods in the synthesis of specific MOFs may be necessary.

## Applications and Mechanisms of MOFs-Based Sensors in Toxins Detection

**FIGURE 2:**
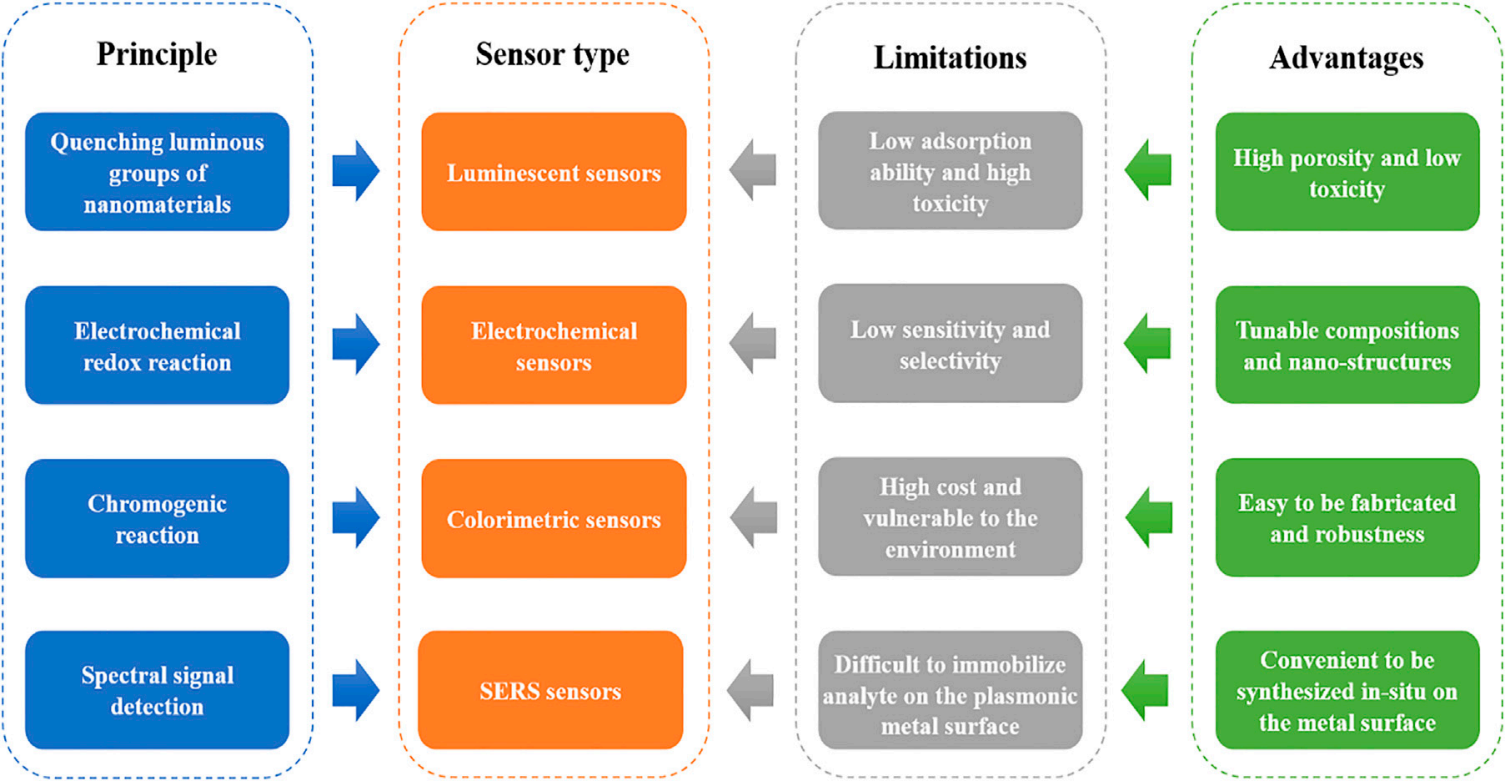
Principle, current limitations of the commonly used food toxins detection sensor types, and the advantages of MOFs in fabricating these sensors.

The rapid detection sensors for food toxins developed with MOFs are mainly based on three fundamental principles: (i) quenching luminous groups of MOFs such as the fluorescence and ultraviolet caused by the conjugated pi groups of ligands and lanthanide ion clusters (Nong et al., 2020) (ii) capturing guest molecules by activated groups of MOFs or encapsulated constituents with further reactions (redox reaction and chromogenic reaction) and (iii) enhancing the spectral signals of target analytes located close to the plasmonic nanoparticles or nanostructured surfaces (Yan et al., 2021; Chio et al., 2022). Therefore, the MOFs-based sensors employed in detecting food toxins can be divided into luminescent, electrochemical, colorimetric, and SERS sensors. Sensitivity and selectivity are two vital factors utilized to measure the performances of these sensors. The porous nature of MOFs can adsorb and pre-concentrate the analytes in complex sample matrices, increasing the possibility of host-guest interactions and further improving the sensitivity of the sensors as mentioned above (Kreno et al., 2012; Hu et al., 2014). For achieving higher selectivity, researchers generally tune the MOFs pore sizes in terms of the sizes of particular food toxin molecules. This section discusses the applications of MOFs in the detection sensors, along with the major limitations of each sensor and the roles of MOFs in overcoming these shortcomings (Figure 2).

Luminescent sensors utilizing the change in fluorescence caused by sensor-analyte interactions are promising in detecting food toxins due to their high sensitivity, rapid response, and simple operation. Most of them employ organic dyes as sensor materials, while the application of MOFs has been emerging recently. For instance, (Bhardwaj et al., 2017) reported the design of an iron-based amine-functionalized NH_2_-MIL-53 and its application for highly sensitive and specific detection of *staphylococcus aureus* (*S. aureus*) in food. With the employment of the NH_2_-MIL-53-based sensor, the photoluminescence intensity would change with respect to varing bacterial concentrations, which offered an easy and feasible way for the detection of *S. aureus*. In the study by (Yue et al., 2022), highly fluorescent fusiform Al-MOF nanosheets were fabricated and used to construct a novel fluorescent sensor for nitrofuran detection in milk. The recovery and relative standard deviation (RSD) reached 88.14%–126.21% and 2.85%–8.13%, respectively. (Peng et al., 2021) used the strategy of functionalizing luminescent guest ion Tb^3+^ within non-luminescent Zr-MOFs to provide luminescence. The Tb^3+^-functionalized Zr-MOF probe demonstrated many attractive sensing properties toward thiabendazoles (TBZ) in oranges, such as broad linear range (0–80 μM), high selectivity, low LOD (0.271 μM), and rapid response time (less than 1 min). The research of MOF-based luminescent sensors toward the chemo-sensing of food toxins has made remarkable progress in recent years. Compared with traditional luminescent dyes and materials, the structures of MOFs in the sensors can be easily modified to meet the need to detect different food toxins. With the continuing improvement of researchers’ ability to design MOFs-based luminescent sensors for targeted applications, it is no doubt that exciting results will emerge soon.

Electrochemical sensors, based on the analyte’s redox reactions in the electrochemical system, offer an attractive alternative for detecting food toxins, as the advantages of low cost, short response time, convenient operation, and easy miniaturization (Wang et al., 2015). The employment of MOFs in electrochemical sensors enables the analytic signal amplification effect and leads to higher sensitivities. For example, (Wang et al., 2022a) modified a molecularly imprinted electrochemical sensor (MIES) with Cu-MOF and Ti_3_C_2_T_x_. The MIES showed shorter response time and high selectivity in determining hygromycin B (a kind of aminoglycosides antibiotic). Its rapidity, selectivity, and sensitivity were attributed to the synergistic signal amplification effect brought about by Cu-MOF and MXene. (Niu et al., 2021) applied an advanced hollow NiCo_2_O_4_@C composite derived from bimetallic Co/Ni-MOF to an electrochemical sensor. The hollow structure accelerated the transport of mass and electrons during the redox reaction, which is beneficial to the detection of furazolidone (FZD) and chloramphenicol (CAP) in milk and honey. In summary, MOFs’ large specific surface area, good absorbability, and tailorable chemical characteristics enable them to transfer electrons to the detected analytes fast and, consequently, illustrate quick responses during detection.

In colorimetric sensors, the optical properties of the nanomaterials change as they interact with the target analyte. The chromogenic reaction of optical nanomaterials is induced, and the amounts of target analytes can be calculated by measuring the color change during the detection process (Fang et al., 2018). Enzymes, which have high catalytic efficiency and target specificity, are broadly used in colorimetric sensors. However, the drawbacks of high cost and vulnerability to harsh environments prevent them from wider applications. Recently, MOFs have been considered ideal alternatives to enzymes in colorimetric sensors. (Tong et al., 2020) fabricated a bioenzyme-free sensitive colorimetric sensor derived from polyoxometalates-based MOFs (POMOFs) for the first time. The nanocomposites showed high sensitivity (1–60 μM), fast response (10 min), and low LOD (2.07 μM) in the detection of citric acid. (Chen et al., 2018) utilized the enhanced peroxidase nanozyme activity of a nickel MOF (Ni-MOF) 2D nanosheet for colorimetric detection of H_2_O_2_. The Ni-MOF nanosheet possessed a higher affinity for H_2_O_2_ than sensors with common natural enzymes, resulting in higher selectivity. Accordingly, as MOFs have similar properties with natural enzymes, they are easy to fabricate and cheap and satisfy most of the toxins’ detection needs, hence drawing attention. However, intense efforts are necessary to invent novel MOFs that can change color within visual range and overcome the problem of utilising additional optical instruments.

Due to the outstanding performance in ultra-sensitivity, SERS sensors based on the spectral technique have been extensively applied to detect food toxins in recent years. Generally, noble plasmon nanoparticles (mainly Au and Ag) are employed as SERS substrates in which an enhanced electromagnetic field could be generated. To improve the performance of SERS, the analyte is required to be limited or close to metal surfaces (Jiang et al., 2015). Herein, the *in-situ* growth of MOFs offers an excellent choice to fabricate high-performance SERS sensors. Wang’s group (Wang et al., 2022b) successfully constructed a versatile self-supporting SERS chip (S-MOF@Au). The S-MOF was formed *in-situ* by directly using a flexible Ni sheet as both the metal source and scaffold. The large amount of SERS “hot spots” in the oriented layered S-MOF@Au resulted in a strong electromagnetic field for Raman amplification with superior reproducibility. This SERS sensor showed low LOD (1.0 × 10^−9^ M for TA, 8.0 × 10^−11^ M for CP, 6.4 × 10^−11^ M for IDP and 5.0 × 10^−12^ M for CV) and satisfactory recoveries (96.2–125.1%) in dectecting tartrazine (TA), chloramphenicol (CP), imidacloprid (IDP) and crystal violet (CV) in food samples (milk, orange juice, and fishes). (Xu et al., 2022) used an *in-situ* growth method to obtain the FP/Ag/ZIF-8-based SERS substrate composed of filter paper (FP), silver nanoparticles (AgNPs), and zeolitic imidazolate framework (ZIF-8) film. The synergy between the components achieved the SERS detection of 4-ATP down to 10^−11^ M with high reproducibility. The flexible FP/Ag/ZIF-8-based sensor showed high sensitivity in detecting pesticide thiram in real lake water, peach juice, and apple pel. These facile SERS substrates were supposed to be promising in food analysis and environmental monitoring.

The flexible synthesis methods, large specific surface area, tunable composition, and mesoporous structures make MOFs effective probe nanomaterials in various food toxins sensors. Considering the promising potential, MOFs-based sensors will see broader applications shortly.

## Conclusion and Outlook

Recently, MOFs have exhibited exciting potential for rapid, efficient, and safe detection of food toxins. MOFs are now drawing increasing attention in food safety, and the focus is directed towards fabricating novel MOFs to detect various food toxins effectively. The advantages of MOFs include high specific surface area, porous structures, and tunable compositions, enabling them to demonstrate outstanding performance in detecting food toxins. MOFs are expected to be widely employed in food safety applications. However, several challenges remain that should be seriously considered at present.

i) To date, many novel, sophisticated synthesis methods (SiO_2_ coated/mixed strategy, polymers coated/electrospun method, surfactants assisted strategy, etc.), for MOFs have been developed to meet the need to detect food toxins. However, the current synthesis methods can hardly ensure the precise fabrication of MOFs. Therefore, it is important to pay more attention to the synthesis of high-quality MOFs.
ii) The composition of MOFs vary widely, and the functions of MOFs can be adjusted according to the food toxins detected. Still, the screening of high-performance MOFs often means the consumption of workforce and resources. To save the costs in time and money, it is reasonable to realize MOFs screening through artificial intelligence and machine learning methods, which are based on density functional theory, finite elements analysis, etc.
iii) MOFs and their derivates have shown superiority in detecting food toxins over other nanomaterials. However, further improvements in combining MOFs with enzymes, aptamers, and antibodies, need to be made to achieve the goal of small molecules analysis.
